# Laboratory animal models for esophageal cancer

**DOI:** 10.14202/vetworld.2016.1229-1232

**Published:** 2016-11-11

**Authors:** Dhanya Venugopalan Nair, A. Gopala Reddy

**Affiliations:** Department of Veterinary Pharmacology and Toxicology, College of Veterinary Science, Rajendranagar, Hyderabad, Telangana, India

**Keywords:** animal model, cancer, esophagus, mice, rat

## Abstract

The incidence of esophageal cancer is rapidly increasing especially in developing countries. The major risk factors include unhealthy lifestyle practices such as alcohol consumption, smoking, and chewing tobacco to name a few. Diagnosis at an advanced stage and poor prognosis make esophageal cancer one of the most lethal diseases. These factors have urged further research in understanding the pathophysiology of the disease. Animal models not only aid in understanding the molecular pathogenesis of esophageal cancer but also help in developing therapeutic interventions for the disease. This review throws light on the various recent laboratory animal models for esophageal cancer.

## Introduction

Cancer is a fatal disease characterized by abnormal proliferation of cells as a result of genetic mutation [1]. Unlike benign tumors that have a good prognosis, malignant tumors or what is referred to as cancer, causes metastasis affecting other organs, eventually leading to death [2]. Hence, cancer is given its due importance in experimental research.

Esophageal cancer stands eighth among the most common cancers in the world [3]. It is also the sixth leading cause of cancer-related mortality in the world [4]. Developing countries make up for more than 80% of the total clinical cases of esophageal cancer [5]. It is often diagnosed at a very advanced stage and thus carries poor prognosis [6,7]. The incidence of the disease varies depending on geographical location [3], ethnicity [3], and socioeconomic status [8]. Consumption of alcohol, tobacco chewing, and smoking are important risk factors that contribute to this disease [9]. The two major subtypes of esophageal cancer are esophageal squamous cell carcinoma and esophageal adenocarcinoma, the former being more common [10]. However, the incidence of adenocarcinoma is also at rise in patients with Barrett’s esophagus due to excessive use of proton pump inhibitors [11,12].

Animal models play an important role in cancer research owing to their ability to generate a better understanding about various molecular mechanisms of metastasis and tumor host interaction [13-15]. They are also necessary to elucidate the pathophysiology of various diseases and indirectly facilitate the development of novel anticancer interventions. The present review article briefs the recent laboratory animal models of esophageal cancer.

## Laboratory Animal Models in Esophageal Cancer

### Rat (*Rattus norvegicus*)

#### Rat reflux model

The reflux model is appropriate to study esophageal adenocarcinoma [15]. However, few recent studies have demonstrated the development of squamous cell carcinoma in this model [16,17]. The rat is considered as suitable for this model because of its larger size as compared to mouse and similar pathophysiology of esophageal adenocarcinoma as compared to that in humans. The EDA model, i.e., esophagoduodenal anastomosis with total gastrectomy is a preferred surgical procedure to establish this model [16]. The gastroesophageal junction and the gastroduodenal junction are ligated. Further, the distal esophagus is transected 2 mm above the gastroesophageal ligature, and the proximal duodenum is transected 3 mm distal to the pyloric area. Following this a gastrectomy is performed and both the ends of the esophagus and duodenum are anastomosed. The maximum duration required for the development of this model is approximately 40 weeks [16].

#### Orthotopic xenograft implantation model

This model is useful to study esophageal squamous cell carcinoma. The human esophageal squamous cell carcinoma cells such as TE6, TE10, or TT are injected subcutaneously into cervical esophagus of athymic nude rats. The model develops in 6 weeks [18]. One of the disadvantages of this model is the inability to study the tumor host relationship factors as the animals are immunodeficient.

#### Chemical induction

N-nitrosomethylbenzylamine (NMBA) is a common mutagen that can induce esophageal squamous cell carcinoma in rats. Rats are subcutaneously injected with NMBA at the rate of 3.5 mg/kg once a week for 5 weeks [19]. The maximum duration for the development of this model is 15 weeks [19].

### Mouse (*Mus musculus*)

#### Transgenic mouse models

The advantage with transgenic animals is the short time period required for the development of the model, as the animals are genetically modified. The Epstein–Barr virus ED-L2, an early lytic cycle promoter that targets the cyclin D1 in a transgenic mouse model leading to dysplasia and premalignant condition in the esophagus is the most commonly used method to induce esophageal squamous cell carcinoma [20,21]. A genetic combination of the above-mentioned L2-cyclin D1 mice and a P53 (tumor suppressor gene) deficient mice is a comparatively better animal model for esophageal squamous cell carcinoma [21,22]. Esophageal adenocarcinoma can be induced by insertion of interleukin-1β cDNA into the Epstein–Barr virus ED-L2 leading to columnar metaplasia similar to that observed in Barrett’s esophagitis [23].

#### Subcutaneous xenograft model

This is the most common and comparatively old method developed to induce esophageal cancer. Human esophageal cancer cell lines are injected subcutaneously into dorsolateral flanks of immunodeficient mice [24]. Several human esophageal cell lines are available such as KYSE-270, KYSE-30, and KYSE-70 that produce esophageal squamous cell carcinoma and OACM5.1 C, SK-GT-4, etc., that produce adenocarcinoma. This model is comparatively easy to develop, however, as the induction is done in an immunodeficient mice, critical components of tumor-host interactions, and metastasis cannot be studied. Moreover, cell lines lack tumor heterogenecity [25]. The minimum time required for development of tumor is 4 weeks [24].

#### Orthotopic xenograft model

This is a recent method different from subcutaneous xenograft model and helpful in studying tumor progression and metastasis. Unlike, the previous model, this model is developed by surgical transplantation of histologically intact human cancer fragments. Initially, the esophageal cell lines are subcutaneously injected into the immunodeficient mice. After development of the tumor, it is aseptically resected, fragmented and fragments of approximately 1 mm^3^ are surgically implanted into the esophageal serosa of another set of immunodeficient mice [26]. However, analysis of tumor growth in this model requires modern diagnostic techniques such as bioluminescent imaging [25]. Transfection with luciferase reporter gene is a commonly used noninvasive technique used to analyze tumor progression, wherein tumor cell lines are transfected ith pLVX-luciferase plasmids before subcutaneous injection into immunodeficient mice [26,27]. The animals are further subjected to bioluminescent imaging during the experiment at regular intervals to study tumor progression. A bioluminescence imaging system with a novel luciferin analogue has recently been developed with enhanced penetration capacity to target deeper tissues [28].

#### Patient derived xenograft model

This model is similar to subcutaneous xenograft model except for the fact that the immunodeficient mice in this model are injected with tumor biopsy cells obtained from clinical cases of esophageal cancer [29,30]. The advantage with this model is that the heterogeneity of the tumor is maintained [25]. This animal model takes on an average 3 months to develop [29,30]. However, the inability to study tumor host interaction due to the use of immunodeficient mice also applies to this animal model.

#### Chemical induction in transgenic model

4-nitroquinoline 1-oxide (4-NQO), NMBA, deoxycholate, and N-methyl-N-nitrosourea are some of the mutagens used to induce esophageal squamous cell carcinoma. Of these 4-NQO appears to be the preferred mutagen [31,32]. It takes on an average 16 weeks for the model to develop post 8 weeks of 4-NQO treatment. 4-NQO in conjunction with a genetically modified/transgenic mouse model stands one of the best animal models to study esophageal squamous cell carcinoma. A miR-31 (miR-31 is an upregulated microRNAs in squamous cell carcinoma) overexpressed transgenic model [33], Nrf2-knockout model [34], and p53 mutated model [35] are some of the few preferred transgenic animal models to which 4-NQO can be administered to induce esophageal squamous cell carcinoma.

### Rabbit (*Oryctolagus cuniculus*)

#### Induction of tumor cells by surgical technique

This model is appropriate to develop esophageal squamous cell carcinoma. The VX2 tumor cell suspension is surgically introduced from the esophageal tunica adventitia into the submucosal layer or muscular layer of the cervical esophagus [36].

#### Endoscopic implantation model

This model is similar to surgical induction of VX2 tumor cell suspension except that the induction is performed with an endoscope. The tumors are harvested from rabbits that were previously implanted intramuscularly with VX2 tumor cells. The harvest is performed after 3 weeks of implantation [37]. Fragments from the harvested tumor are introduced with an endoscope into the submucosa of the esophagus [37]. Both the surgical and endoscopic implantation of VX2 cancer cell lines produce different pathological features and effectively mimic human esophageal squamous cell carcinoma [38].

### Guinea pig (*Cavia porcellus*)

#### Gastroesophageal reflux model

This model is appropriate to study esophageal adenocarcinoma. A gastroesophageal reflux model is developed by perfusion of esophagus with HCl containing 1 g/L pepsin for 20 min/day [39,40]. The animals develop inflammation as a result of the acid, which in turn triggers adenocarcinoma mimicking that of Barrett’s esophagus [25].

### Hamster (*Mesocricetus auratus*)

#### Benzo[a]pyrene (BP) induced model

Although hamsters are widely used as a model for development of oral cancer, they have been neglected species in the development of esophageal cancer. Induction of esophageal cancer by instillation of BP in the esophagus twice weekly for 5 weeks is a good animal model to study esophageal squamous cell carcinoma [41].

## Conclusion

The recent increase in the incidence of esophageal cancer, delayed diagnosis and poor prognosis are the major factors that demand better therapeutic interventions for this disease. As an efficient animal model is the first stepping stone toward developing an effective drug, this review was pursued. However, during the process of review, it was observed that there was paucity of efficient animal models. Although evident progress had been made in the development of transgenic models, certain important aspects such as the mechanisms related to tumor host relationship, effect of environment on the development of the disease and response of the disease to various therapeutic agents could not be completely elucidated due to the use of immunodeficient animals. Hence, a holistic understanding of the disease is yet to be achieved, which is only possible on overcoming these challenges. Researchers should also focus on identifying new molecular targets. These targets are to be are validated by *in vitro* methods and the genes that regulate these molecular targets are to be identified. Subsequently, genetically modified preclinical models are to be developed. Some novel molecular targets for esophageal cancer have already been reported in previous studies [42,43]. These targets need to be taken to the next level of preclinical validation to develop efficient animal models.

## Authors’ Contributions

DV and AG have participated in the discussion, draft, and revision of the manuscript. DV and AG have read and approved the final manuscript.

## References

[1] Washington M.C, Leaver D (2016). Principles and Practice of Radiation Therapy.

[2] Kerr D.J, Haller D.G, Van de Velde C.J.H, Baumann M (2012). Oxford Textbook of Oncology.

[3] Naufal R, Mohamed E, Michael K, Amjid R (2015). Current management of oesophageal cancer. Br. J. Med. Pract.

[4] Ferlay J, Shin H.R, Bray F, Forman D, Mathers C, Parkin D.M (2010). Estimates of worldwide burden of cancer in 2008: GLOBOCAN 2008. Int. J. Cancer.

[5] Ohashi S, Miyamoto S, Kikuchi O, Goto T, Amanuma Y, Muto M (2015). Recent advances from basic and clinical studies of esophageal squamous Cell carcinoma. Gastroenterology.

[6] Manish A.S (2015). Update on metastatic gastric and esophageal cancers. J. Clin. Oncol.

[7] Dreikhausen L, Blank S, Sisic L, Heger U, Weichert W, Jäger D, Bruckner T, Giese N, Grenacher L, Falk C, Ott K, Schmidt T (2015). Association of angiogenic factors with prognosis in esophageal cancer. BMC Cancer.

[8] Dar N.A, Shah I.A, Bhat G.A, Makhdoomi M.A, Iqbal B, Rafiq R, Nisar I, Bhat A.B, Nabi S, Masood A, Shah S.A, Lone M.M, Zargar S.A, Islami F, Boffetta P (2013). Socioeconomic status and esophageal squamous cell carcinoma risk in Kashmir India. Cancer Sci.

[9] IARC Working Group (2012). Personal habits and indoor combustions. IARC Monographs on the Evaluation of Carcinogenic Risks to Humans.

[10] Philip R.T, Christian C.A, Sanford M.D (2013). Squamous dysplasia –The precursor lesion for esophageal squamous cell carcinoma. Cancer Epidemiol. Biomarkers Prev.

[11] Thrift A.P, Whiteman D.C (2012). The incidence of esophageal adenocarcinoma continues to rise: Analysis of period and birth cohort effects on recent trends. Ann. Oncol.

[12] Greene C.L, Worrell S.G, De M.T.R (2015). Rat reflux model of esophageal cancer and its implication in human disease. Ann. Surg.

[13] Hibberd C, Cossigny D.A.F, Quan G.M.Y (2013). Animal cancer models of skeletal metastasis. Cancer Growth Metastasis.

[14] Saxena M, Christofori G (2013). Rebuilding cancer metastasis in the mouse. Mol. Oncol.

[15] Ruggeri B.A, Camp F, Miknyoczki S (2013). Animal models of disease: Pre-clinical animal models of cancer and their applications and utility in drug discovery. Biochem. Pharmacol.

[16] Hashimoto N (2012). Expression of COX2 and p53 in rat esophageal cancer induced by reflux of duodenal contents. ISRN Gastroenterology.

[17] Kumagai H, Mukaisho K.I, Sugihara H, Miwa K, Yamamoto G, Hattori T (2004). Thioproline inhibits development of esophageal adenocarcinoma induced by gastroduodenal reflux in rats. Carcinogenesis.

[18] Hori T, Yamashita Y, Ohira M, Matsumura Y, Muguruma K, Hirakawa K (2001). A novel orthotopic implantation model of human esophageal carcinoma in nude rats: CD44H mediates cancer cell invasion *in vitro* and *in vivo*. Int. J. Cancer.

[19] Wargovich M.J, Woods C, Eng V.W, Stephens L.C, Gray K (1988). Chemoprevention of N-nitrosomethylbenzylamine-induced oesophageal cancer in rats by the naturally occurring thioether, diallyl sulfide. Cancer Res.

[20] Nakagawa H, Wang T.C, Zukerberg L, Odze R, Togawa K, May G.H, Wilson J, Rustgi A.K (1997). The targeting of the cyclin D1 oncogene by an Epstein-Barr virus promoter in transgenic mice causes dysplasia in the tongue, esophagus and forestomach. Oncogene.

[21] Jenkins T.D, Nakagawa H, Rustgi A.K (1997). The keratinocyte-specific Epstein-Barr virus ED-L2 promoter is regulated by phorbol 12-myristate 13-acetate through two cis-regulatory elements containing e-box and Krüppel-like factor motifs. J. Biol. Chem.

[22] Opitz O.G, Harada H, Suliman Y, Rhoades B, Sharpless N.E, Kent R, Kopelovich L, Nakagawa H, Rustgi A.K (2002). A mouse model of human oral-esophageal cancer. J. Clin. Invest.

[23] Quante M, Bhagat G, Abrams J, Marache F, Good P, Lee M.D, Wang T.C (2012). Bile acid and inflammation activate gastric cardia stem cells in a mouse model of Barrett’s-like metaplasia. Cancer Cell.

[24] Crescenzi M, Persano L, Esposito G, Zulato E, Borsi L, Balza E, Ruol A, Ancona E, Indraccolo S, Amadori A (2011). Vandetanib improves anti-tumor effects of L19mTNFalpha in xenograft models of esophageal cancer. Clin. Cancer Res.

[25] Tétreault M.P (2015). Oesophageal cancer: Insights from mouse models. Cancer Growth Metastasis.

[26] Shuai S, Dong C, Yong C, Jian H, Min G, Kai M, Fang D, Zhi-Hua L, Tian-You W (2014). New orthotopic implantation model of human esophageal squamous cell carcinoma in athymic nude mice. Thorac. Cancer.

[27] Joseph C.Y.I, Josephine M.Y.K, Valen Z.Y, Kwok W.C, Alfred K.L, Simon L, Daniel K.H.T, Maria L.L (2015). A versatile orthotopic nude mouse model for study of esophageal squamous cell carcinoma. BioMed Res. Int.

[28] Takahiro K, Satoshi I, Masahiro K, Shun M, Tetsuya K, Haruki N, Shojiro M, Shinae K (2016). A luciferin analogue generating near-infrared bioluminescence achieves highly sensitive deep-tissue imaging. Nat. Commun.

[29] Xianhua W, Jingchuan Z, Ruheng Z, Jing L.V, Li Z, Xinying S, Guanshan Z, Paul R.G, Songtao X, Shaohua L, Jun H, Yalan L, Chen X, Yunshan T, Liang X, Xiaolu Y, Deming H, Qunsheng J, Yingyong H, Di G (2012). Trastuzumab anti-tumor efficacy in patient-derived esophageal squamous cell carcinoma xenograft (PDECX) mouse models. J. Transl. Med.

[30] Zhang J, Jiang D, Li X, Lv J, Xie L, Zheng L, Gavine P.R, Hu Q, Shi Y, Tan L, Ge D, Xu S, Li L, Zhu L, Hou Y, Wang Q (2014). Establishment and characterization of esophageal squamous cell carcinoma patient-derived xenograft mouse models for preclinical drug discovery. Lab. Invest.

[31] Yang Z, Guan B, Men T, Fujimoto J, Xu X (2013). Comparable molecular alterations in 4-nitroquinoline 1-oxide-induced oral and esophageal cancer in mice and in human esophageal cancer, associated with poor prognosis of patients. In Vivo.

[32] Baba S, Yamada Y, Hatano Y, Miyazaki Y, Mori H, Shibata T, Hara A (2009). Global DNA hypomethylation suppresses squamous carcinogenesis in the tongue and esophagus. Cancer Sci.

[33] Tseng S.H, Yang C.C, Yu E.H, Chang C, Lee Y.S, Liu C.J, Chang K.W, Lin S.C (2015). K14-EGFP-miR-31 transgenic mice have high susceptibility to chemical-induced squamous cell tumorigenesis that is associating with Ku80 repression. Int. J. Cancer.

[34] Akira O, Takafumi S, Masao O, Toshimitsu K, Masayuki Y (2013). Roles of keap1–Nrf2 system in upper aerodigestive tract carcinogenesis. Cancer Prev. Res.

[35] Zhang Z, Wang Y, Yao R, Li J, Lubet R.A, You M (2006). P53 transgenic mice are highly susceptible to 4-nitroquinoline-1-oxide-induced oral cancer. Mol. Cancer Res.

[36] Liu J, Li N, Li L, Li D, Liu K, Zhao L, Tang J, Li L (2013). Local hyperthermia for esophageal cancer in a rabbit tumor model: Magnetic stent hyperthermia versus magnetic fluid hyperthermia. Oncol. Lett.

[37] Jin H, Jinquan S, Guanyin X, Xiang W, Yin Z, Xiaowei T, Zhining F, Yingzhou S, Hanming S, Zhi L (2014). Establishing a rabbit model of malignant esophagostenosis using the endoscopic implantation technique for studies on stent innovation. J. Transl. Med.

[38] Huang J, Zhang Y, Zhong H, Fan Z, Jiang G, Shen Y, Song H, Tao Z, Wang K (2014). Comparison of endoscopic submuscosal implantation vs. Surgical intramuscular implantation of VX2 fragments for establishing a rabbit esophageal tumor model for mimicking human esophageal squamous carcinoma. PLoS ONE.

[39] Yan-Mei C, Ai-Li C, Jian-Pu Z, Hong-Wei W, Yong-Shun S, Chun-Fang L, Bei-Bei Z, Yi W, Sheng-Liang Z, Da-Zheng W (2014). Airway hyper responsiveness induced by repeated esophageal infusion of HCl in guinea pigs. J. Respir. Cell Mol. Biol.

[40] Qinzi L, Lingfei K, Shuna Z, Zhaoshuang Z, Xiaofeng L, Jinou W, Jian K (2010). A novel external esophageal perfusion model for reflux-associated respiratory symptoms. Pathobiology.

[41] Dunham L.J, Sheets R.H (1974). Sheets effects of esophageal constriction on benzo[*a*]pyrene carcinogenesis in hamster esophagus and forestomach. JNCI J. Natl. Cancer Inst.

[42] Zhao J.Y, Wang F, Li Y, Zhang X.B, Yang L, Wang W, Xu H, Liu D.Z, Zhang L.Y (2015). Five miRNAs considered as molecular targets for predicting esophageal cancer. Med. Sci. Monit.

[43] Boone J, Van Hillegersberg R, Offerhaus G.J, Van Diest P.J, Rinkes I.H.B, Kate F.J.T (2009). Targets for molecular therapy in esophageal squamous cell carcinoma: An immunohistochemical analysis. Dis. Esophagus.

